# Comparing the Inhibitory Effect of Kefir Drink and Chlorhexidine Mouthwash against Oral Bacteria in Orthodontic Patients 

**DOI:** 10.30476/dentjods.2024.102609.2371

**Published:** 2025-06-01

**Authors:** Leila Jabbareh, Hodis Ehsani, Hamidreza Goli, Abolfazl Hosseinnataj, Sajjad Ebadian, Melika Mollaei, Tahura Etezadi

**Affiliations:** 1 Dept. of Periodontics, Dental Research Center, Faculty of Dentistry, Mazandaran University of Medical Sciences, Sari, Iran.; 2 Molecular and Cell Biology Research Center, Faculty of Medicine, Mazandaran University of Medical Sciences, Sari, Iran.; 3 Dept. of Biostatistics, Faculty of Health, Mazandaran University of Medical Sciences, Sari, Iran.; 4 Dentist, Dental Research Center, Student Research Committee, Faculty of Dentistry, Mazandaran University of Medical Sciences, Sari, Iran.; 5 Dentistry Student, Dental Research Center, Student Research Committee, Faculty of Dentistry, Mazandaran University of Medical Sciences, Sari, Iran.; 6 Dept. of Orthodontics, Dental Research Center, Faculty of Dentistry, Mazandaran University of Medical Sciences, Sari, Iran.

**Keywords:** Probiotic, Kefir Grains, Orthodontics, Chlorhexidine Hydrochloride, Oral bacteria

## Abstract

**Background:**

The presence of orthodontic appliances in the oral cavity increases the number of cariogenic microorganisms, highlighting the risk of periodontal disease and dental caries. Many approaches can be employed to overcome this problem.

**Purpose:**

This study was conducted to compare the effect of kefir drink with chlorhexidine (CHX) mouthwash on the growth of oral bacteria in patients undergoing orthodontic treatment.

**Materials and Method:**

In this single-blind clinical trial study, 30 candidates for orthodontic treatment were selected and randomly divided into two groups (N=15). The intervention group received 100 ml of Kefir drink twice daily, while the control group used 10 ml of 0.2% mouthwash for 20 days. Microbial sampling was done three times including before intervention, immediately after intervention, and 20 days after intervention. Data were analyzed using SPSS software V.23 with a significance level of less than 0.05.

**Results:**

The findings suggested that the number of bacteria in the CHX group was significantly higher than in the intervention group (*p*= 0.003). However, no significant difference was observed between the two groups after 20 days (*p*= 0.148). Furthermore, the number of bacteria decreased significantly in both groups over time.

**Conclusion:**

Both CHX and Kefir have antibacterial properties against oral bacteria.

## Introduction

Orthodontic treatment has become increasingly popular owing to advantages in improving facial beauty, smile, chewing, and self-esteem. However, Food impaction can occur around the bands, brackets, and wires used in patients receiving fixed orthodontic treatment. The proliferation of germs is facilitated by the difficulty of maintaining oral and dental hygiene, which leads to dental caries [ 1 - 2 ]. Since Streptococcus mutans (S. *mutans*) is also the cause of primary decay, reducing it may decrease decay [ 3 ].

When combined with mechanical teeth cleaning techniques, applying chemical and herbal substances enhances their effectiveness and successfully reduces microbial plaque. Nowadays, mouthwashes are among the most significant antibacterial compounds available for reducing oral organisms [ 4 - 5 ].

Chlorhexidine (CHX) is one of the most widely used mouthwashes, which has been introduced as a gold standard to compare other anti-microbial agents [ 6 ]. Notwithstanding its advantageous antibacterial properties, poor taste, taste changes, and the possibility of changing the color of oral tissues have all contributed to consumer dissatisfaction [ 7 ].

Many approaches have been employed, such as antibiotic and antimicrobial therapies, but their effectiveness is only visible when they are used regularly. These drawbacks have led to the idea of using alternative methods such as probiotics [ 8 ]. Probiotics are described as "live microorganisms that, when consumed in sufficient quantities, confer health on the host" by the World Health Organization [ 9 ]. Probiotics counteract the acid produced by sugar metabolism and function as antagonists of the bacteria that cause dental cavities [ 10 ].

Probiotics, which are found in kefir grains in a symbiotic connection with other microbes, are recognized in this fermented beverage. This drink is rich in vitamin K, B1, B2, calcium, folic acid, and amino acids, which support overall health and control a variety of diseases. Apart from therapeutic advantages, consumers do not consider Kefir as a medicinal beverage owing to its tasty flavor and accessibility [ 11 ].

It is vital to eradicate caries-inducing microbes during orthodontic treatment due to the heightened risk of periodontal disease and dental caries. Furthermore, considering the side effects of CHX mouthwash and the paucity of research on Kefir drink, the current investigation was conducted to compare the inhibitory effect of Kefir drink and CHX mouthwash against oral bacteria in patients undergoing orthodontic treatment. 

## Materials and Method

### Study design

This single-blinded clinical trial obtained ethical approval from Mazandaran University of Medical Sciences (IR.MAZUMS.REC.1401.14269) and the protocol was submitted to the Iranian Registry of Clinical Trials (IRCT20200905048620N1). An informed consent was obtained from the patients after explaining the purpose and methodology of the study.

The study population consisted of the patients who were referred to a private clinic in Sari during 2022-2023, using the census sampling method. The inclusion criteria were systematically healthy orthodontic patients who were more than 15 years old. Patients with periodontitis, allergic reactions, and a history of antibiotic or corticosteroid use over the last three months were excluded [ 12 ]. The sample size was calculated to be 30 patients (15 controls, 15 interventions) based on Ghasempour *et al*.’s [ 11 ] investigation and considering 20% dropout:

n=(z1-α/2+z1-β)2*(σ12+σ22)(μ1-μ2)2 α=0.01 β=0.1

The patients were randomly categorized into two groups (N=15): the CHX group (control) and the Kefir group (intervention). The control group received daily 10 ml of 0.2% CHX mouthwash (Iran Najo Pharmaceutical Company) for 20 days. They had to rinse their mouth with 5 ml of mouthwash twice a day (morning and night) for 30 seconds.

The Kefir group was instructed to use 100 ml of Kefir drink every 12 hours for 20 days. For producing the Kefir mouthwash, milk with 1.5 percent fat (Pegah, Iran) was boiled and cooled down at room temperature. Subsequently, Kefir grains were added and fermented at 250C for 48 hours.

### Microbial test

Both solutions were stored in bottles with the same color and shape to blind the experiment. All participants received training about brushing their teeth from a senior dental student who was not aware of their study group. The instructions regarding the use of the solutions were given to the patients in separate envelopes by a senior dental student. The examiner and the patients were not aware of the contents of the containers. The patients were asked to avoid drinking and eating for an hour after using the substances [ 11 ].

Three samples were obtained from the patients as follows: the first sampling was performed two months after the bracket bonding around the left upper premolar and canine teeth, and the rings were placed in normal saline. The second sampling was performed following the first sampling (on the same day), and the patients were asked to rinse their mouths with CHX for 30 seconds or consume the Kefir drink (based on their group). Subsequently, the elastic rings on the right side of the patients in the same areas were removed immediately after being washed and placed in normal saline. The third sampling was performed 20 days after the first and second sampling. During this procedure, the elastic rings of the right canine and the upper premolar were removed and placed in normal saline [ 13 ]. 

The orthodontic rings were placed in normal saline and transferred to the microbiology laboratory for colony counting. First, the serial dilutions from the original samples were prepared and cultured in 100 microliters of Mitis salivarius agar culture medium. Then, the dilutions were incubated for 48 hours at 37°C and 10% CO2. The number of colonies was counted and compared in both groups [ 13 ]. All evaluations and removal of elastomeric rings were performed by an orthodontist.

### Statistical analysis 

In this study, descriptive indices such as mean, standard deviation, frequency, and percentage were used. Independent t-tests and chi-square tests were used to compare demographic variables in two groups. Non-parametric Mann-Whitney and Friedman tests were also used to compare the number of bacteria in the groups over time. A generalized estimating equation (GEE) test was performed to investigate the effect of group and time on the number of bacteria simultaneously. Data were analyzed using SPSS software version 23 and the significance level was considered less than 0.05. 

## Results

**Figure 1:**
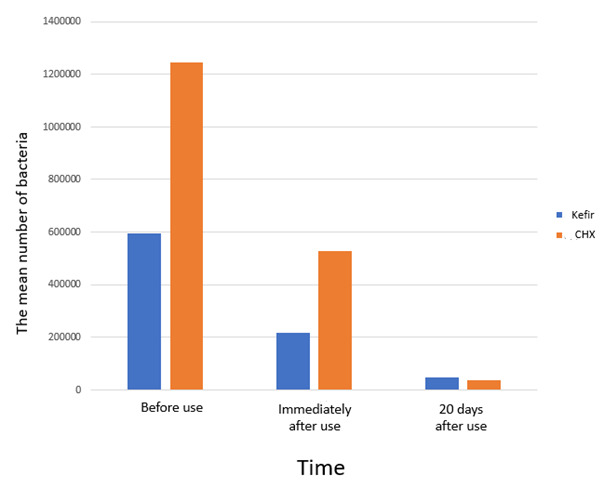
The number of bacteria in the intervention and control groups

The current clinical trial included 30 orthodontic patients of which 19 (63.3%) were women and 11 (36.7%) were men. The mean age of the participants was 20.70± 4.96 years old. As observed in Table 1 and Figure 1, there was no significant difference in terms of age and sex between the groups (*p*> 0.05). 

**Table 1:** Demographic features of the participants in the intervention and control groups

Variable		Kefir	CHX	Total	*p* Value
Age*		19.5±	21.3±	20.4±	0.177
Mean ± SD		47.87	93.63	70.96	
Sex**	Woman	11(73.3)	8 (53.3)	19 (63.3)	0.450
N (%)	Man	4(26.7)	7 (46.7)	11 (36.7)	

* Independent t-test ** Chi-square test CHX= Chlorhexidine, SD= standard deviation

The number of bacteria at different times was compared according to the type of solution. The Mann-Whitney test showed that there was a significant difference in the number of bacteria observed between the two groups before and immediately after using the mouthwashes, and that the number of bacteria in the CHX group was significantly higher than the intervention group (*p*= 0.003). However, no significant difference was observed between the two groups after 20 days (*p*= 0.148). Moreover, the number of bacteria was compared over time and the results revealed that the number of bacteria decreased significantly in both groups (Table 2).

**Table 2:** Comparing the number of bacteria in the intervention and control groups

Time	Kefir			CHX			*p* Value*	NTT
	Mean	SD	Mean rank	Mean	SD	Mean rank		
Before use	596666.7	606649.7	11.7	1246666.7	1243765.4	19.3	0.016	2.77
Immediately after use	217666.7	342568.4	10.8	490213.7	490213.7	20.2	0.003	2.53
20 days later	47733.3	58495.6	13.2	29472.7	27472.7	17.3	0.148	11.99
*p* Value**	0.001>			0.001>			-	-

* Mann-Whitney test ** Freidman test CHX= Chlorhexidine SD= standard deviation NTT= Number-Needed-to-Treat

Findings from the GEE test in Table 3 discovered that group and time had a significant effect on the number of bacteria. On average, the number of bacteria in the Kefir drink was 316511.1 less than CHX mouthwash (*p*= 0.014). Moreover, the number of bacteria immediately after use and 20 days after using the solution was 549500 and 878666.7 less than before use, respectively. 

**Table 3:** Comparing the effect of group and time on the number of bacteria

Variable		Regression coefficient	SD	Confidence interval 95%	*p* Value	Partial Eta Squared
Group	Kefir	-316511.1	128741.2	(-568839.2, -64183.1)	0.014	0.11
	CHX	Reference				
Time	Before use	Reference				0.48
	Immediately after use	-549500	157675.1	(-858537.5, -240462.5)	0.001>	
	20 days after	-878666.7	157675.1	(-1187704.2, -569629.2)	0.001>	

* Mann-Whitney test** Freidman test CHX= Chlorhexidine, SD= standard deviation

## Discussion

Orthodontic appliances are susceptible to deterioration and colonization of acidic bacteria that accumulate plaque. Demineralization happens when the pH of dental plaque lowers during orthodontic treatment as a result of bacteria fermenting carbohydrates in the diet [ 14 ]. CHX mouthwash has been extensively studied and suggested to be the most effective anti-plaque and anti- gingivitis agent. Nevertheless, it has several detrimental local side effects, including brown discoloration of the teeth and oral mucosa, taste disturbance, and in severe cases, sensitivity, and parotid duct narrowing [ 15 ]. Therefore, alternative anti-plaque substitutes such as probiotics are developed as a result of these negative aspects. 

Probiotics can prevent the growth of biofilms, cellular adhesion, and colonization in their pathogenic surroundings [ 16 ]. The antiplaque property of probiotics can be observed in several methods such as diminishing bacterial adhesion to the tooth surface, inhibiting the growth and proliferation of microorganisms on the tooth surface, preventing the formation of intercellular plaque matrix, altering plaque biochemistry to minimize the formation of cytotoxic products, and modifying plaque ecology to a less pathogenic flora [ 17 ].

This clinical trial examined the inhibitory effect of Kefir drink and CHX mouthwash against oral bacteria on 30 orthodontic patients and discovered that the average number of bacteria isolated from the elastic rings of the patients was reduced in both groups, and the reduction was significantly greater in the Kefir group compared to the CHX group. 

Similarly, a study by Widyarman *et al*. [ 18 ] suggested that daily consumption of probiotic yogurt reduced the quantity of S. *mutans* in the saliva of patients under fixed orthodontic treatment. Moreover, Alp *et al*. [ 14 ] assessed the effect of daily consumption of Kefir on the microbial colonization in the saliva of orthodontic patients and revealed that Kefir could reduce the number of S. *mutans* and Lactobacillus in the saliva.

Other studies have used saliva to determine the amount of S. *mutans* in the oral cavity [ 19 - 20 ]. The main disadvantage of using saliva is that it represents the total quantity of microorganisms in the oral cavity. Nonetheless, tongue and caries surfaces contain various types of organisms that are not specific to the tooth surface. Some studies have shown different results concerning the number of salivary S. *mutans* in dental plaque [ 21 - 22 ]. One of the strengths of the current study was using the orthodontic elastic rings instead of saliva to assess microbial accumulation, which has more reliable findings since they are in close contact with the tooth surface and provide suitable retention for microbial growth. A study by Näse *et al*. [ 23 ] suggested the anticariogenic effect of the long-term use of Lactobacillus rhamnosus Gorbach-Goldin (LGG) milk. It should be noted that milk is rapidly washed; however, Kefir has a more viscous nature compared to milk and can persist in the oral cavity [ 24 ]. Additionally, Shah *et al*. [ 17 ] compared probiotics with CHX and revealed that probiotics were as effective as CHX in plaque control. Moreover, the probiotic group showed more improvement in gingival indices and plaque indices compared to the CHX group. Their findings suggested that probiotic mouthwash is effective in reducing plaque accumulation and gingivitis. On the other hand, Qasempour *et al*. [ 25 ] conducted a study with the aim of comparing the effect of the probiotic Kefir yogurt drink, 0.2% CHX, and 0.2% sodium fluoride on S. *mutans*. This in vitro study showed that kefir could inhibit S. *mutans* more than sodium fluoride and less than CHX. The difference between the findings can be due to the laboratory nature of their study, which could not stimulate the variables in the oral cavity.

Numerous organic substances possess the potential to prevent dental caries caused by fixed orthodontic treatment; however, their applicability is contingent upon several other considerations. When utilizing these components, it is crucial to keep in mind their effectiveness, stability, flavor, aroma, and cost [ 26 ]. Kefir is one of the few natural substances, which has all these characteristics. Based on the findings of the current and previous research, Kefir has antibacterial properties and it can be considered as a suitable alternative for long-term use in patients undergoing orthodontic treatment. 

This study only assessed the inhibitory effect of Kefir on oral bacteria; although this bacterium is the primary cause of caries and microbial plaque, other species are also involved. Furthermore, confounding variables including people's diets, which were not controllable in this study, are among its other drawbacks. 

## Conclusion

Following the intervention, the mean quantity of oral bacteria isolated from orthodontic rings in both groups was notably decreased. The study's findings suggest that Kefir and CHX have comparable antibacterial properties. Kefir is more advantageous and suggested in long-term therapies like orthodontics since it is a good substitute for CHX mouthwash.
